# The Neural Mechanisms of the Effect of Spontaneous Insight on Re-Solution: An ERP Study

**DOI:** 10.3390/jintelligence11010010

**Published:** 2023-01-03

**Authors:** Jie Chen, Ke Zhang, Xiumin Du, Junmiao Pan, Jing Luo

**Affiliations:** 1 College of Education, Hebei University, No. 180 of Wusi East Road, Baoding 071002, China; 2 Beijing Key Laboratory of Learning and Cognition, School of Psychology, Capital Normal University, Beijing 100048, China

**Keywords:** insight, memory, re-solution, ERP

## Abstract

The insight memory advantage refers to the situation in which memory performance could be improved by solving a problem with an Aha experience. In re-solution tests and recognition tests, studies demonstrate an insight memory advantage by spontaneous insight or induced insight. For the re-solution test, the neural mechanisms of the effect of induced insight were studied by the fMRI technique. However, the neural mechanisms of the effect of insight on re-solution in the temporal dimension were not known. The neural mechanisms of the effect of spontaneous insight on re-solution were not known. In the present study, we use the compound remote-associated (CRA) task to reveal the neural mechanisms of the effect of spontaneous insight on re-solution by the event-related potentials (ERPs) technique. The 25 participants were asked to solve a series of Chinese verbal CRA tasks and then perform a re-solution test 1 day later. Our results indicated that the solution with the Aha experience evoked a larger N400 in the early solution phase and a more negative wave in the late solution phase than the solution with no Aha experience. In the re-solution phase, items with an Aha during the solution phase were re-solved better with higher Aha rates than items with no Aha. In the re-solution phase, compared with items with no Aha, items with an Aha during the solution phase evoked a larger positive ERP in the 250 to 350 ms time window in the early phase, and a more negative deflection before the response (−900 to −800 ms) in the later phase. In one word, spontaneous insight during the solution phase could promote re-solution and elicit ERP deflection in the re-solution phase.

## 1. Introduction

Most people have experienced the insight phenomenon where the solution to a complex problem pops up unexpectedly. To be specific, insight is a sudden understanding of a new relationship among known stimuli, accompanied by the Aha experience, which includes emotional and cognitive components (Kizilirmak et al. 2016b; Shen et al. 2018). Since Köhler (1917) first proposed the concept of insight, more and more researchers have carried out detailed studies from the perspectives of cognition and emotion (Danek and Wiley 2020; Kizilirmak et al. 2016b; Shen et al. 2017). On the one hand, during insightful problem solving, sudden changes in the problem representation and restructuring process occur, which are considered to be the cognitive components of insight (Bilalić et al. 2021; Danek and Wiley 2020; Knoblich et al. 1999; Ohlsson 1992, 2011). On the other hand, the Aha experience is the key to distinguishing insight from non-insight, as well as from surprise, certainty, happiness and so on (Danek et al. 2014; Kaplan and Simon 1990; Shen et al. 2016; Sternberg and Davidson 1995; Topolinski and Reber 2010). However, recent studies found that insightful solutions do not necessarily involve impasses and a restructuring process, especially in CRA problem solving (Becker et al. 2021; Cranford and Moss 2012). For instance, Cranford and Moss reported that immediate insight (i.e., the first candidate solution generated was the correct solution) might not include the cognitive process of traditional insight, but it was also accompanied by the Aha experience. In addition, insight mainly occurs in two ways: induced insight (occurs when answers are presented to participants) and spontaneous insight (occurs when participants solve problems independently) (Auble et al. 1979; Danek et al. 2013, 2014; Du et al. 2017; Jung-Beeman et al. 2004; Ludmer et al. 2011; Shen et al. 2018; Tik et al. 2018; Kizilirmak et al. 2016b).

Many studies have clarified the mnemonic effect of insight solution by means of recognition and recall tests or re-solution tests, which are separately regarded as the direct test or indirect test (Auble et al. 1979; Danek et al. 2013; Kizilirmak et al. 2016b; Köhler 1921; Richardson-Klavehn 2010). In recognition and recall tests, the insightful memory effect was demonstrated (Cui et al. 2021; Kizilirmak et al. 2016b; Shen et al. 2021, 2022). For example, Kizilirmak et al. (2016b) found that items with an Aha experience were recognized better than those without an Aha experience. Shen et al. (2021) investigated ads that could induce insight or not. They found that participants recognized ads with induced insight better than those with non-insight. In addition, Danek and Wiley (2020) used magic tricks as experimental materials and reported that, after a week, solutions with an Aha experience were recalled better than those without an Aha experience.

As an indirect test, the re-solution test also demonstrated an insight memory advantage. At the beginning of the study of insight, Köhler (1921) found that apes re-solved problems more efficiently (the RT of the re-solution was shorter than that of the first attempt) when they first solved such problems by insight. Kizilirmak et al. (2016b) explored the effects of Aha experiences on memory with Mooney images. The results showed that, compared with items solved without an Aha, items solved with an Aha during the study phase were re-solved better during the test phase. This study also showed that although both the recognition test and the re-solution test are memory tests, they are different from each other. Regarding the retrieval strategy, the recognition test is mainly involved with direct retrieval. However, the re-solution process may be more complex, involved with not only direct retrieval, but also procedural retrieval (Roussel et al. 2002; Uittenhove et al. 2016; Tagart et al. 2015). Furthermore, on the level of consciousness, the recognition test mainly involved voluntary, controlled retrieval, whereas the re-solution test mainly involved involuntary, automatic retrieval (Richardson-Klavehn 2010).

With the development of cognitive neuroscience, it is necessary to study the neural mechanisms of psychological processes. Recent studies have shown the neural mechanisms of insight memory advantage in recognition tests by spontaneous insight and induced insight. For instance, Kizilirmak et al. (2016a) used the fMRI technique to investigate the underlying neural processes of induced insight on memory in recognition tests. They observed that activation of the amygdala was associated with successful recognition of items with an Aha experience by using an induced insight paradigm. Considering the difference between spontaneous insight and induced insight, the neural mechanisms of the influence of spontaneous insight on memory was explored. Cui et al. (2021) and his colleagues found a more positive N400 and LPC amplitude induced by spontaneous solutions with an Aha experience in the recognition test phase than those with no Aha experience. 

The neural mechanisms of the effect of induced insight on re-solution were explored by the fMRI technique. Ludmer et al. (2011) has found that amygdala activation during the study phase could be used to predict the re-solution performance of items with an Aha experience a week later. However, in the temporal dimension, the neural mechanisms of the effect of insight on re-solution are not known. At behavioral and neurophysiological levels, spontaneous insight was different from induced insight (Rothmaler et al. 2017). The neural mechanisms of the effect of spontaneous insight on re-solution are not known. Therefore, in the present study, we mainly investigated the effect of spontaneous insight on re-solution from the neural level by using the ERPs technique. 

Previous studies showed that, in the problem-solving process, the N400 amplitude was larger for insight items than non-insight items (Li et al. 2013; Luo et al. 2011; Qiu et al. 2008). The N400 for insight problem solving was suggested to reflect breaking the prior problem representation and establishing new connections (Li et al. 2013; Qiu et al. 2006; Zhou et al. 2018). In recent years, researchers began to explore the differences between insight and non-insight by response-locked ERPs (Cui et al. 2021; Kizilirmak et al. 2021). They paid attention to the electrophysiological differences within about 1000 ms before the response of the solution that was generated with an Aha experience or not. For example, our previous study found that items with an Aha induced a more negative wave than those with no Aha at −800 to −400 ms before the solution was generated (Cui et al. 2021). The response-locked ERPs was suggested to reflect the experienced differences between insight and non-insight (Cui et al. 2021). In addition, during creative problem solving based on previous experience, the N400 from about 300 to 500 ms might reflect an integration process, and the LPC from about 500 to 800 ms was responsible for the elaborate processing of the input information, both based on memory (Luo et al. 2011; Zhou et al. 2020; Zhao et al. 2017; Rataj et al. 2018).

In this study, we used the compound remote associates (CRA) task to investigate the neural mechanisms of the effect of spontaneous insight on re-solution by the ERPs technique. We investigated the N400 component in the early solution phase and the ERP deflection in the late solution phase; in the re-solution phase, we investigated the insight memory advantage in the early re-solution phase and the mean amplitude at −1000 to −400 ms before the solution in the late re-solution phase.

## 2. Materials and Methods

### 2.1. Participants

The twenty-eight subjects who volunteered to participate (14 women, 14 men, *M*_age_ = 21.32, SD = 2.05) in the experiment were postgraduates and undergraduates of Hebei University. All subjects were native Chinese speakers and right-handed. Before participating in the experiment, they were told that the experiment was divided into two phases with an interval of one day, and they signed an informed consent form. Because the data of one participant were poor and two participants did not follow the instructions, the data of three participants were excluded. Consequently, only the data of 25 participants were analyzed. Each participant who completed the experiment received a reward of 40 yuan.

### 2.2. Design and Materials

A within-subjects design was employed in this experiment. The independent variable is Aha or not. We selected 320 Chinese CRA items as experimental stimuli from a CRA corpus (Du et al. 2017). For the CRA, each item contained three clue words and a solution word. The solution word could be combined with each clue word to form a two-character word. For example, if the clue words were “yu” (渔)/“wan” (湾)”/” kou” (口), participants needed to obtain the solution word “gang”(港) to solve the problem. The position of the solution word could be before or after the clue words in the two-character words: “gang”(港) after “yu” (渔) forms a two-character word, “yu gang” (渔港); before “wan” (湾), it forms a two-character word, “gang wan” (港湾); and before “kou” (口), it forms a two-character word, “gang kou” (港口). There was only one correct solution word for each CRA problem (Du et al. 2017). A large number of previous studies have proved that these items could induce the Aha experience well and have good stability (Bowden and Jung-Beeman 2003; Jung-Beeman et al. 2004). We informed the participants of the detailed explanation of the insight and ensured that they fully understood it before the experiment. The Chinese introduction of characteristics of insightful solution was as follows:

“The definition of insight is sudden and obvious problem-solving process. You may not know how you think of the answer in the process of solving the problem. But at some point, the answer suddenly appears, and you don’t need to confirm it again to think it should be the right answer.”

There are 160 items in the solution phase and 320 items in the re-solution phase (160 new items and 160 old items).

### 2.3. Procedure

#### 2.3.1. Solution Phase

**Figure 1:**
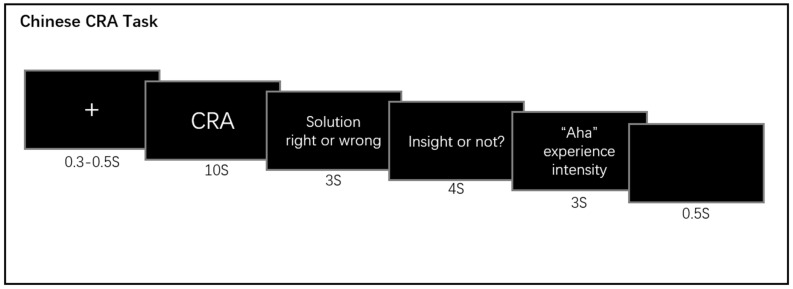
Experimental procedure: the solution test and re-solution test both are CRA task.

In the solution phase (Figure 1), for each trial, a fixation “+” was presented in the middle of the screen for 300 to 500 ms first. Moreover, 300 to 500 ms temporal jitters were used before the stimulus presentation. After that, the CRA was presented for 10 s and allowed to be solved by participants. Participants were required to press the space key immediately once they obtained the solution. Then, the screen showed the correct solution for 3 s, which allowed the participants to honestly judge whether their solution was correct. The participants pressed the “f” key if their solution was correct and the “j” key if their solution was incorrect. If the participant did not solve the problem within 10 s, the screen still showed the solution, which allowed participants to watch for 3 s. Finally, participants needed to report whether they had an Aha experience (“f” key: Aha; “j” key: no Aha) within 4 s, and they also rated the Aha experience intensity (a 5-point scale was used) for 3 s. There were 160 items in the solution phase, and participants were allowed to rest every time they completed 40 items.

#### 2.3.2. Re-Solution Phase

The re-solution phase was about one day after the solution phase. It should be noted that we did not inform the participants of the specific content of the test for the next day in advance. The experimental procedure in the re-solution phase was exactly the same as that in the solution phase, but we added 160 new items as interference items.

### 2.4. ERP Recording and Analysis

Electroencephalography (EEG) was recorded at a 32-channel electrical geodesic net (Brain Product), with an amplifier (lower cut-off frequency 0.01 Hz, upper cut-off frequency 100 Hz, 500 Hz sampling rate). According to the international 10–20 system, the electrodes were positioned on an elastic cap. Each interelectrode impedance was kept below 5 KΩ during recording. The EEG data were filtered offline using IIR filters (0.01–35 Hz). Rejection for eye movements were done offline by Ocular Correction ICA. Trials with eye movement deflection or another incorrect behavioral response were excluded from the ERP averaging (exceeding ± 100 microvolts at any electrode, based on a 200 ms prestimulus baseline in the early phase or a 300~500 ms after-response baseline in the later phase). 

In the solution phase, each trial consisted of two epochs. The EEG for the early solution phase was epoched offline from 200 ms before the stimulus onset to 2000 ms after the stimulus onset, with a 200 ms pre-stimulus baseline. The EEG for the later solution phase was epoched offline from 1000 ms before the response to 500 ms after the response, with a 300~500 ms after-response baseline. The following nine electrode points, both in the solution phase and the re-solution phase, were chosen for statistical analysis: F3, Fz, F4, C3, Cz, C4, P3, Pz and P4 electrodes. In the solution phase, we mainly analyzed the N400 (400~600 ms) in the early solution phase and the mean amplitude from −1000 to −400 ms before the solution in the late solution phase. We conducted a three-way repeated measures ANOVA with the factors solution type (2 levels: Aha, no Aha), region (3 levels: front—Fz, F3, F4; central—Cz, C3, C4; parietal—Pz, P3, P4) and laterality (3 levels: left—F3, C3, P4; middle—Fz, Cz, Pz; right—F4, C4, P4) in the solution phase. In addition, we mainly analyzed the N400 (from 400 to 600 ms after the stimulus onset) and the LPC (from 600 to 800 ms after the stimulus onset) in the early re-solution phase and the time window of −1000 to −400 ms before the solution in the late re-solution phase. We conducted a three-way repeated measures ANOVA with the factors solution type (2 levels: old, new), region (3 levels: front, central, parietal) and laterality (3 levels: left, middle, right) and a three-way repeated measures ANOVA with the factors solution type (2 levels: Aha, no Aha), region (3 levels: front, central, parietal) and laterality (3 levels: left, middle, right) in the re-solution phase. According to the Greenhouse–Geisser method, we corrected the deviation of the *p* value of all the analyses.

## 3. Results

In the present study, the items analyzed in the solution phase were also correctly solved in the re-solution phase. 

### 3.1. Behavioral Performance

#### 3.1.1. Behavioral Performance in the Solution Phase

In the solution phase, a total of 44.99% (SD = 9.55) of the CRA items were solved correctly by the participants. For all correctly solved items, items with an Aha experience accounted for 54.77% (SD = 10.66), and items with no Aha experience accounted for 44.71% (SD = 10.79). For the reaction times (RTs) of the solution, no significant difference was found between the items with an Aha experience (*M* = 4.99 s, SD = 1.04 s) and the items with no Aha experience (*M* = 4.54 s, SD = 0.90 s), *t* (24) = 1.474, *p* = 0.15.

#### 3.1.2. Behavioral Performance in the Re-Solution Phase

We analyzed memory performance on items with an Aha and items with no Aha by the successful re-solution rate and the RTs of re-solution. In the re-solution phase, participants correctly solved 84.96% (SD = 7.13) of the old items and 55.40% (SD = 9.58) of the new items. A significant difference was found between the re-solution rate of the old and new conditions, *t* (24) = 14.238, *p* < 0.001. Old items were easier to solve than new items. In addition, we observed a difference in RTs of re-solution between the old (*M* = 3.17 s, SD = 0.84) and new (*M* = 4.01 s, SD = 0.83 s) conditions, *t* (24) = −11.635, *p* < 0.001. Re-solving the old items was faster than re-solving the new items. 

In addition, we further analyzed the spontaneous solution with an Aha and that with no Aha contained in the old condition. The insight memory advantage was obtained: items with an Aha (86.90 ± 6.43) had a better re-solution rate than items with no Aha (82.42 ± 12.00), *t* (24) = 2.08, *p* < 0.05. However, the differences in RTs of the re-solution were not significant between the solutions with an Aha condition (*M* = 3.29 s, SD = 0.94) and the solutions with no Aha condition (*M* = 3.16 s, SD = 0.72), *t* (24) = 1.096, *p* = 0.284. 

Furthermore, in this phase we analyzed the Aha rates of those items with and without an Aha experience in the solution phase. We found that the Aha rates of the items with an Aha (*M* = 37.43 %, SD = 24.56) during the solution phase were higher than those with no Aha (*M* = 30.16 %, SD = 21.98) during the solution phase, *t* (24) = 2.308, *p* < 0.05.

### 3.2. Electrophysiological Scalp Data

#### 3.2.1. Electrophysiological Scalp Data in the Solution Phase

**Figure 2:**
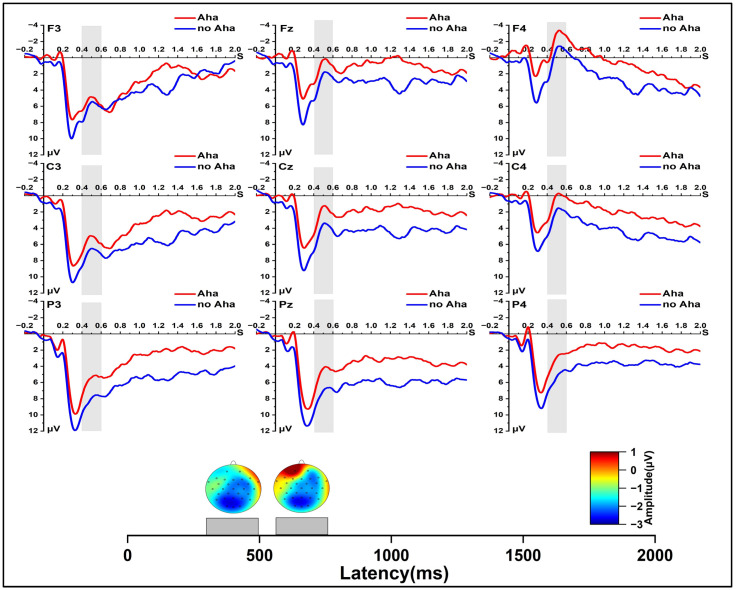
Grand-average ERPs from all selected channels for Aha and no Aha conditions and spherical-spline interpolated scalp distributions of the solution type (Aha minus no Aha) in 400~500 ms and 500~600 ms time windows.

In the early solution phase, as shown in Figure 2, we found a significant main effect of solution type, *F* (1, 24) = 11.872, *p* < 0.01, *η_p_*^2^ = 0.331; a significant main effect of region, *F* (2, 48) = 18.241, *p* < 0.001, *η_p_*^2^ = 0.432; and a significant main effect of laterality at 400 to 500 ms, *F* (2, 48) = 24.315, *p* < 0.001, *η_p_*^2^ = 0.503. The result of the interaction revealed a significant region × laterality interaction effect, *F* (4, 96) = 9.175, *p* < 0.001, *η_p_*^2^ = 0.276. At 500 to 600 ms, the repeated measures ANOVA also showed a significant main effect of solution type, *F* (1, 24) = 11.596, *p* < 0.01, *η_p_*^2^ = 0.326; a significant main effect of region, *F* (2, 48) = 18.426, *p* < 0.001, *η_p_*^2^ = 0.434; and a significant main effect of laterality, *F* (2, 48) = 33.958, *p* < 0.001, *η_p_*^2^ = 0.586. The interaction of laterality and region was significant, *F* (4, 96) = 14.068, *p* < 0.001, *η_p_*^2^ = 0.370. Further pairwise comparison showed the N400 (400~600 ms) amplitude was larger in the Aha condition than the no Aha condition, *p* < 0.01; *p* < 0.01.

**Figure 3:**
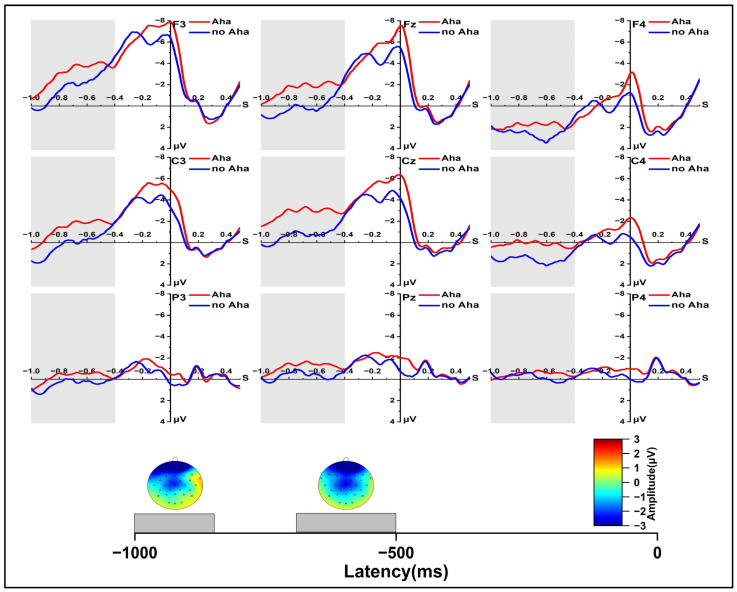
Grand-average ERPs from all selected channels for Aha and no Aha conditions and spherical-spline interpolated scalp distributions of the solution type (Aha minus no Aha) in −1000 to −700 ms and −700 to −500 ms time windows.

In the late solution phase (Figure 3), for the −500 to −400 ms time window, the repeated measures ANOVA showed a significant main effect for laterality, *F* (2, 48) = 14.022, *p* < 0.001, *η_p_*^2^ = 0.369, and a significant interaction of laterality and region, *F* (4, 96) = 30.500, *p* < 0.001, *η_p_*^2^ = 0.560. At −700 to −500 ms, the repeated measures ANOVA showed significant main effects for solution type, *F* (1, 24) = 4.347, *p* < 0.05, *η_p_*^2^ = 0.153, and for laterality, *F* (2, 48) = 11.925, *p* < 0.001, *η_p_*^2^ = 0.332. A more negative wave for the Aha condition than the no Aha condition was found, *p* < 0.05. There was a significant interaction of laterality and region, *F* (4, 96) = 28.829, *p* < 0.001, *η_p_*^2^ = 0.546. At −1000 to −700 ms, and a significant main effect of laterality was found, *F* (2, 48) = 5.091, *p* < 0.05, *η_p_*^2^ = 0.175. Significant effects were also found in the two-dimensional interaction between solution type and region, as well as laterality and region, *F* (2, 48) = 3.59, *p* < 0.05, *η_p_*^2^ = 0.130; *F* (4, 96) = 18.134, *p* < 0.001, *η_p_*^2^ = 0.430. Further simple effect analysis showed that, compared with the no Aha condition, the Aha condition evoked a more negative ERP in the central region, *p* < 0.05.

#### 3.2.2. Electrophysiological Scalp Data in the Re-Solution Phase

**Figure 4:**
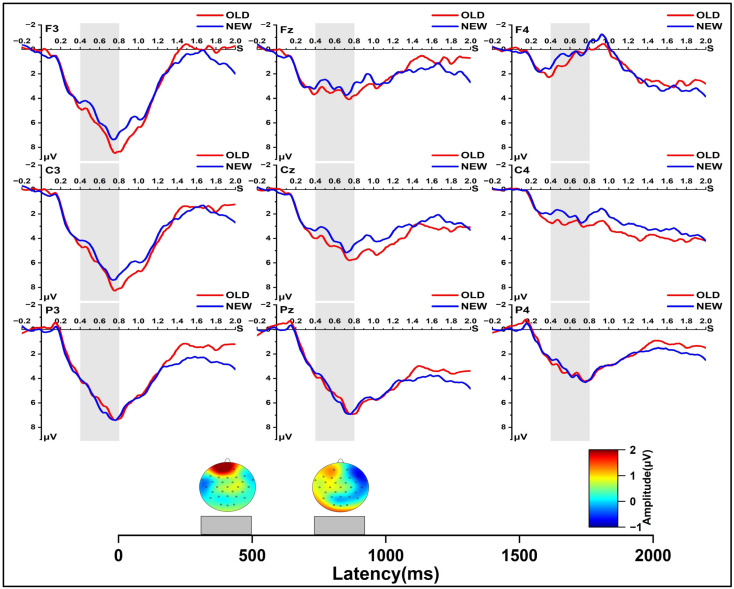
Grand-average ERPs from all selected channels for Old and New items and spherical-spline interpolated scalp distributions of the solution type (Old minus New) in 400~500 ms and 700~800 ms time windows.

In the early re-solution phase for the old and new conditions (Figure 4), at 400~500 ms, we found a significant main effect of solution type, *F* (1, 24) = 5.131, *p* < 0.05, *η_p_*^2^ = 0.176, and a significant main effect of laterality, *F* (2, 48) = 24.679, *p* < 0.001, *η_p_*^2^ = 0.507. Further pairwise comparison indicated that old items resulted in a significantly higher positive wave than the new items, *p* < 0.05. There was an interaction of laterality and region, *F* (4, 96) = 4.873, *p* < 0.01, *η_p_*^2^ = 0.169. At 500~600 ms, we found a significant main effect of laterality, *F* (2, 48) = 27.647, *p* < 0.001, *η_p_*^2^ = 0.535, and a significant main effect of region, *F* (2, 48) = 4.444, *p* < 0.05, *η_p_*^2^ = 0.156. We also found a marginally significant interaction effect between solution type and region, *F* (2, 48) = 3.410, *p* = 0.059, *η_p_*^2^ = 0.124, and a significant interaction effect between region and laterality, *F* (2, 48) = 7.785, *p* = 0.001, *η_p_*^2^ = 0.245. Further simple effect analysis showed that in the central region, old items resulted in a significantly higher positive wave than the new items, *p* < 0.05. At 600~700 ms, we found a significant main effect of laterality, *F* (2, 48) = 33.298, *p* < 0.001, *η_p_*^2^ = 0.581, and a significant main effect of region, *F* (2, 48) = 6.928, *p* < 0.01, *η_p_*^2^ = 0.224. In addition, we found a marginally significant solution type × region interaction effect, *F* (2, 48) = 3.190, *p* = 0.068, *η_p_*^2^ = 0.117, and a significant region × laterality interaction effect, *F* (4, 96) = 12.409, *p* < 0.001, *η_p_*^2^ = 0.341. At 700~800 ms, we found a significant main effect of laterality, *F* (2, 48) = 34.324, *p* < 0.001, *η_p_*^2^ = 0.589, and a significant main effect of region, *F* (2, 48) = 8.189, *p* < 0.01, *η_p_*^2^ = 0.254. We found a marginally significant solution type × region interaction effect, *F* (2, 48) = 3.184, *p* = 0.068, *η_p_*^2^ = 0.117, and a significant region × laterality interaction effect, *F* (4, 96) = 18.259, *p* < 0.001, *η_p_*^2^ = 0.432. In the central region, old items resulted in a significantly higher positive wave than the new items, *p* < 0.05. 

**Figure 5:**
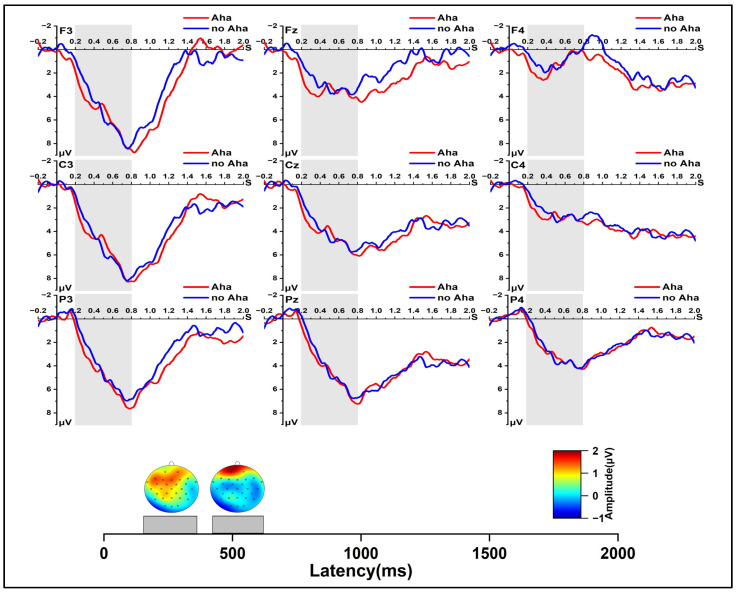
Grand-average ERPs from all selected channels for Aha and no Aha conditions and spherical-spline interpolated scalp distributions of the solution type (Aha minus no Aha) in 250~350 m and 400~500 ms time windows.

In the early re-solution phase for the Aha and no Aha conditions, as shown in Figure 5, at 250~300 ms, the results revealed a significant main effect of solution type, *F* (1, 24) = 5.784, *p* < 0.05, *η_p_*^2^ = 0.194, a significant main effect of region, *F* (2, 48) = 8.504, *p* < 0.01, *η_p_*^2^ = 0.262, and a significant main effect of laterality, *F* (2, 48) = 14.153, *p* < 0.001, *η_p_*^2^ = 0.371. At 300 to 350 ms, a marginally significant main effect of solution type was observed, *F* (1, 24) = 3.646, *p* = 0.068, *η_p_*^2^ = 0.132, and a significant main effect of laterality was observed, *F* (2, 48) = 13.934, *p* < 0.001, *η_p_*^2^ = 0.367. Further simple effect analysis showed that the Aha condition evoked a larger P300 amplitude than the no Aha condition at 250 to 350 ms, *p* < 0.05; *p* = 0.068. Significant effects were found in the two-dimensional interaction between region and laterality, *F* (4, 96) = 2.595, *p* = 0.068, *η_p_*^2^ = 0.098. At 400 to 500 ms, we found a significant main effect of laterality, *F* (2, 48) = 16.320, *p* < 0.001, *η_p_*^2^ = 0.405. In addition, a significant effect was also found in the two-dimensional interaction between region and laterality, *F* (4, 96) = 5.343, *p* < 0.01, *η_p_*^2^ = 0.182. A similar result was observed at 500 to 600 ms. In the 600~700 ms and 700~800 ms time windows, we found a significant main effect of region, *F* (2, 48) = 4.183, *p* = 0.042, *η_p_*^2^ = 0.148; *F* (2, 48) = 5.345, *p* = 0.021, *η_p_*^2^ = 0.182, a significant main effect of laterality, *F* (2, 48) = 28.375, *p* < 0.001, *η_p_*^2^ = 0.542; *F* (2, 48) = 31.001, *p* < 0.001, *η_p_*^2^ = 0.564, and a significant interaction effect of laterality × region, *F* (4, 96) = 13.608, *p* < 0.001, *η_p_*^2^ = 0.362; *F* (4, 96) = 19.151, *p* < 0.001, *η_p_*^2^ = 0.444.

**Figure 6:**
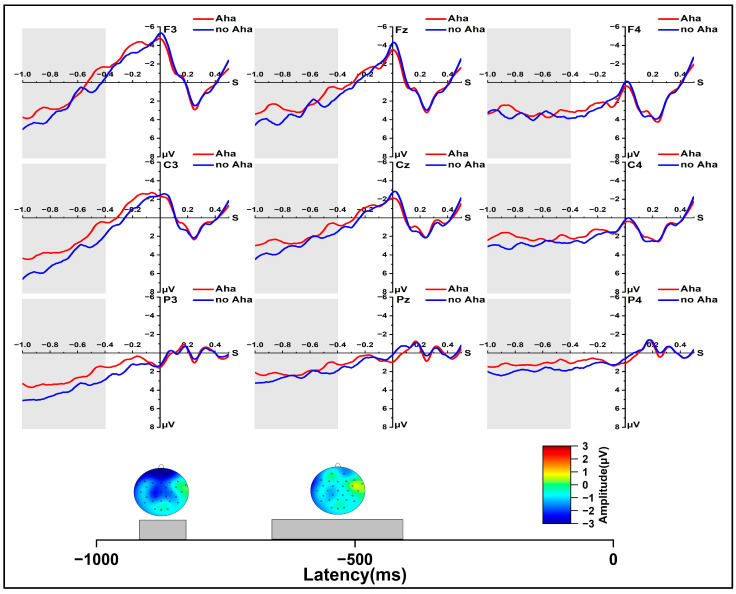
Grand-average ERPs from all selected channels for Aha and no Aha conditions and spherical-spline interpolated scalp distributions of the solution type (Aha minus no Aha) in −900 to −800 ms and −800 to −400 ms time windows.

We further analyzed the difference between the Aha and no Aha conditions in the late re-solution phase (Figure 6). In the time window of −800 to −400 ms, we found a significant interaction effect between laterality and region, *F* (4, 96) = 9.762, *p* < 0.001, *η_p_*^2^ = 0.289. At −900~−800 ms, we found a significant main effect of solution type, *F* (1, 24) = 5.027, *p* < 0.05, *η_p_*^2^ = 0.173. Further pairwise comparison showed a more negative wave for the Aha condition than the no Aha condition, *p* < 0.05. A significant main effect of laterality was observed, *F* (2, 48) = 4.999, *p* < 0.05, *η_p_*^2^ = 0.172. A significant interaction effect of region × laterality was observed, *F* (4, 96) = 3.762, *p* < 0.05, *η_p_*^2^ = 0.136. At −1000~−900 ms, we found a significant main effect of laterality, *F* (2, 48) = 7.181, *p* < 0.01, *η_p_*^2^ = 0.230. 

## 4. Discussion

In the present study, we used the CRA task to investigate the neural mechanisms of the effect of spontaneous insight on re-solution by the ERPs technique. We found: (1) the solution with an Aha experience induced a more negative N400 in the early solution phase and a more negative wave in the late solution phase than the solution without an Aha experience; (2) compared with items without an Aha experience, items with an Aha experience during the solution phase were re-solved better with higher Aha rates in the re-solution phase; (3) items with an Aha experience during the solution phase induced a more positive waveform in the early re-solution phase (250 to 350 ms after stimulus onset) and a more negative waveform in the late re-solution phase (−900 to −800 ms before response) than items without an Aha experience; (4) compared with old items, new items induced more negative waveforms in the N400 component in the re-solution phase. To the best of our knowledge, this is the first ERP study to investigate neural mechanisms of spontaneous insight on re-solution from the temporal dimension.

### 4.1. Behavioral Findings

Our results indicated that, compared with no-Aha items, items with an Aha experience were re-solved better; these results demonstrated an insight memory advantage. This result was consistent with previous findings, in which items with an Aha experience were re-solved better than items without an Aha experience in the CRA test (Kizilirmak et al. 2016b). In addition, no significant difference was found between the RTs of solutions with an Aha experience and those of solutions without an Aha experience in the solution phase; thus, the influence of RTs in the solution phase on re-solution performance could be excluded.

Our results indicated that the Aha rates of items with an Aha experience during the solution phase were higher than items without an Aha experience during the solution phase in the re-solution phase. To our knowledge, this is the first time such a result has been found. Combining the results of the behavior data in the re-solution rate, we found that, although the items with an Aha experience during the solution phase were easier to solve in the re-solution phase, participants still reported a greater Aha experience for them. One possible explanation for these results might be that items with an Aha experience during the solution phase were more likely to be re-solved immediately, accompanied with an Aha experience in the re-solution phase. Cranford and Moss (2012) reported that there were two different types of insight during CRA problem solving, including immediate solutions with an Aha experience, in which the first candidate solution generated was the correct solution, and non-immediate solutions with an Aha experience, in which the first candidate solution was incorrect. Immediate solutions with an Aha experience might not involve impasses and a restructuring process. In this case, participants might report an Aha experience simply because the solution was suddenly obtained with the help of an insight memory advantage, even though these processes did not require any impasses or restructuring. The speculation on this result was also supported by our ERP results, and we will discuss it further in the next part.

### 4.2. ERP Findings

In the early solution phase, our results indicated that solutions with an Aha experience induced a larger amplitude in the N400 component than solutions with no Aha experience. This result is consistent with previous findings (Luo et al. 2011; Mai et al. 2004; Shen et al. 2013; Zhang et al. 2011). Mai et al. (2004) found that items with an Aha experience evoked a more negative wave around 380 ms when using riddle problems. Shen et al. (2013) also found that, compared with non-insight solutions, a more negative wave was observed in the 320~550 ms after stimulus. Previous studies suggested N400 reflected that the process of insightful problem solving involved breaking the prior problem’s representation and establishing new connections (Luo et al. 2013; Mai et al. 2004).

In the late solution phase, our results showed that, compared with solutions with no Aha experience, solutions with an Aha experience evoked a negative wave with a larger amplitude −700 to −500 ms before the solution response. This is in accordance with our previous study, in which Cui et al. (2021) the negative wave found −800~−400 ms before the response was larger in solutions with an Aha experience than in solutions with no Aha experience. Thus, in the late solution phase, there may be essential electrophysiological differences between insight and non-insight (Cui et al. 2021; Jung-Beeman et al. 2004; Rothmaler et al. 2017). In contrast to the early solution phase, this time window (−700 to −500 ms before the solution response) existed in a solution flash moment, accompanied by the occurrence of an Aha experience in the traditional sense (Weisberg 2013; Sternberg and Davidson 1995). Zhao et al. (2013) have found that, only in the late phase, the activity in the amygdala, which is related to the Aha experience, was stronger for insight solutions than non-insight solutions. In addition, Jung-Beeman et al. (2004) found that insight solutions induced high-frequency (gamma-band) neural activity 300 ms before the solution response. Previous studies suggested that the negative wave in this time window (−1000 to −400 ms before the solution response) might be related to the Aha experience (Cui et al. 2021).

In the re-solution phase, our results indicated that the new items evoked a more negative N400(400 to 500 ms) than old items. Therefore, our results showed that the old items might induce a better integration process between the information in memory and problem solving with the help from the experience in the solution phase rather than new items. Suggestions of recent studies have indicated that N400 reflects the difficulty of integration during creative problem solving, as the integration process involves a connection between problems and information in memory (Luo et al. 2011, 2013).

Our results showed no electrophysiological differences between the Aha condition and the no Aha condition for the N400 effect (400~600 ms) and the LPC effect (600~800 ms), and items with an Aha experience during the solution phase evoked a larger P300 in the time window of 250~350 ms in the early re-solution phase. Some studies have found that the degree of the familiarity effect was aroused approximately 250 ms after stimulus onset, and maybe even earlier (Diana et al. 2005; Friedman 2007; Nessler et al. 2005). The present study found a significant difference between items with an Aha experience and those with no Aha experience in the re-solution test within the time window of 250~350 ms, which involves familiarity. In addition, some researchers suggested that increasing the confidence level of decisions correlated with an increase in P300 (Twomey et al. 2015; Rungratsameetaweemana et al. 2018; Squires et al. 1975). In our study, the larger P300 in Aha conditions as opposed to no-Aha conditions might suggest that participants were more confident identifying items with an Aha experience during the solution phase for old items in order to better solve those problems later. To our knowledge, we are the first to discover this effect of the re-solution test.

Our results indicated that, in the late re-solution phase (−900 to −800 ms before response), items that elicited an Aha experience in the solution phase evoked a lager negative amplitude than items with no Aha experience in the solution phase. Consistent with the solution phase, the process of re-solution may also involve an Aha experience. This result was similar with the result in the late solution phase (−1000 to −400 ms before response). The response-locked ERPs were suggested to reflect the experience differences between insight and non-insight in the solution phase at −1000 ms to −400 ms before the response from which the solution was generated (Cui et al. 2021). Thus, in our present study, the more negative wave induced by items with an Aha experience during the solution phase might be associated with a stronger experience in the re-solution process.

With regard to our behavior results, the different Aha rates in the re-solution phase also revealed the experience differences in the re-solution phase between items with an Aha during the solution phase and items with no Aha during the solution phase. One possible explanation for these results comes from the re-solution strategy. Several studies have demonstrated that procedural and direct retrieval were the strategies used to solve the problems (Roussel et al. 2002; Uittenhove et al. 2016; Tagart et al. 2015). As a specifical solution process, re-solution also contained the above two strategies. For the strategy of procedural retrieval, participants were willing to use the same method to solve the same problem when the previous problem reappeared (Schillemans et al. 2012). However, because of the insight memory advantage, participants consciously used previous problem-solving strategies, and the cognitive conflict induced by the insight occurring in the search process of the problem re-solving may have been reduced. For the strategy of direct retrial, some studies suggested that the same emotional experience would be aroused again when people perceived and encoded the same emotional information (Danker and Anderson 2010; Smith et al. 2004). In addition, previous research has claimed that the memory of an emotional experience for events was easy to retrieve and lasting (Ledoux 1996). Especially in a recent study about the source memory of insight, Du et al. (2017) reported that participants had better source memory for Aha experiences than no-Aha experience. Thus, in the re-solution phase, items with an Aha experience during the solution phase were more likely to produce an Aha experience than items with no Aha experience during the solution phase. In conclusion, in the late re-solution phase, although the cognitive conflict of the items with an Aha experience during the solution phase was reduced in the early re-solution phase (N400), these items still evoked a greater Aha experience than items with no Aha experience during the solution phase, which might reflect the transformation from non-immediate insight to immediate insight. 

## 5. Limitation

A main limitation of the current study was that the study design was quasi-experimental because whether a CRA problem would trigger an Aha experience for a particular person or not could not be determined randomly during CRA problem solving. In the past, most of the research on insight mainly used objective and subjective methods. The objective method means the experimenter divides the given problems into insight problems and non-insight problems, according to the characteristics of insight cognitive processing. The subjective method means considering whether participants reported an Aha experience based on their own emotional experience after problem solving (Auble et al. 1979; Mednick 1962; Danek et al. 2013). The limitation of the objective method was that some given insight items might not be solved through insight. The subjective method adopted in this study also had some limitations, which mainly came from the understanding of the participants on insight. Especially, whether some immediate solution items with an Aha experience could be regarded as insight items needs to be discuss further. Some researchers suggested that the insightful solution should contain impasses and a restructuring process, while others suggested that an insightful solution might not need to include these processes. Related studies also believed that an Aha experience was the key to distinguishing insight from non-insight (Danek et al. 2014; Kaplan and Simon 1990; Shen et al. 2016; Sternberg and Davidson 1995; Topolinski and Reber 2010), and our study was also mainly based on this view. Therefore, the results of this study might change due to the different methods (objective versus subjective) used and the different criteria for subjective judgment of insight.

Another limitation of this study was that some CRA problems that evoked an Aha experience differed in some (unknown) systematic way from some CRA problems that did not, and this unobserved variable might have driven the observed effects. A previous proposal might help us overcome this limitation (Steindorf and Rummel 2020). Therefore, data were further analyzed using generalized binomial linear mixed-effects modelling (glmer) with crossed random intercepts for Subjects and Items. All models were fit with the lme4 package in R. For the re-solution rate, there was a fixed effect of solution type (*β* = 0.4136, SE = 0.1462, z = 2.830, *p* < 0.01). As expected, items with an Aha experience during the solution phase were more likely to be re-solved than items with no Aha during the solution phase. For Aha rates in the re-solution phase, there was a fixed effect of solution type (*β* = 0.4166, SE = 0.1258, z = 3.311, *p* < 0.001). The result showed that items with an Aha experience during the solution phase were more likely to be re-solved accompanied with an Aha than items with no Aha experience during the solution phase. In future research, we will examine other variables that may affect the experimental results and use multilevel models to explain the results. 

## Data Availability

The data are currently not publicly available due to participant privacy, but they are available from the corresponding author upon reasonable request.
